# Synthesis of a novel photochemical and thermoresponsive diblock biomaterial with end-functionalized zinc porphyrin

**DOI:** 10.3389/fbioe.2023.1268458

**Published:** 2023-12-01

**Authors:** Nannan Qiu, Xinyuan Pan, Ruizhang Hu, Zhenzhen Hui

**Affiliations:** ^1^ College of Chemistry and Materials Engineering, Anhui Science and Technology University, Bengbu, Anhui, China; ^2^ Anhui Province Quartz Sand Purification and Photovoltaic Glass Engineering Research Center, Fengyang, Anhui, China

**Keywords:** zinc porphyrin, diblock copolymer, fluorescence, cis/trans isomerization, photocatalytic activity, thermosensitive property, atom transfer radical polymerization

## Abstract

Porphyrin compound-based photochemical molecules and biomaterials have been synthesized for photosensitivity and bioimaging experiments. However, most porphyrin photosensitizers have limited application in biological environments owing to severe aggregation in aqueous solutions. In the present study, we prepared amphipathic and photosensitive copolymers using zinc porphyrin via consecutive atom transfer-free radical polymerizations (ATRPs) comprising photoresponsive and thermosensitive chain segments. Furthermore, we evaluated the photocatalytic activity of the copolymer for methylene blue (MB) in water.

**Methods:** First, we synthesized a photoresponsive ain segment of poly (6-[4-(4-methoxyphenylazo)phenoxy]hexyl methacrylate) (ZnPor-PAzo); then, ZnPor-PAzo was used as a macroinitiator and was polymerized with *N*-isopropylacrylamide (NIPAM) via ATRPs to obtain a novel photochemical and thermoresponsive diblock biomaterial with end-functionalized zinc porphyrin [(ZnPor-PAzo)–PNIPAMs].

**Results:** The polydispersity index (*M*_w_/*M*_n_) of (ZnPor-PAzo)–PNIPAMs was 1.19–1.32. Furthermore, its photoresponsive and thermosensitive characteristics were comprehensively studied.

**Discussion:** The end-functionalized diblock copolymer (ZnPor-PAzo)–PNIPAM exhibits obvious fluorescence and efficient photocatalytic activity for aqueous MB under visible light.

## 1 Introduction

The basic framework of porphyrins and their derivatives comprises four pyrrolic subunits that are linked by four methyne bridges. Owing to their conjugated macrocyclic structures, porphyrins possess excellent photochemical and photophysical characteristics (Liang et al., 2021). Therefore, porphyrins and their derivatives have widespread applications, including CO_2_-reducing agents (Miyaji and Amao, 2021), light-harvesting materials (Zhang et al., 2003), pharmaceutical biomaterials (Panagiotakis et al., 2022), and photocatalysts (Kechich et al., 2021). However, porphyrins are prone to aggregation and insoluble in water. Therefore, they fail to exhibit photochemical activity in biological environments (Hone et al., 2002). Considering the abovementioned limitations, researchers have attempted to combine porphyrins with hydrophilic polymers or micelles (Ren et al., 2011; Tse et al., 2019). In our previous study, we prepared different end-functionalized poly (*N*-isopropylacrylamide) (PNIPAMs) via atom transfer-free radical polymerization (ATRP) and investigated their photodynamic therapy (Yang et al., 2014) and optical limiting properties (Qiu et al., 2013). Indeed, these end-functionalized polymers with porphyrin not only retained their photochemical and photophysical properties but also possessed water solubility and thermosensitivity owing to the introduction of hydrophilic chain segments. However, we observed that fluorescence intensity (Qiu et al., 2013; Yang et al., 2014) or reverse saturation absorption (Qiu et al., 2013) have a certain degree of diminished in intensity. This may be because the PNIPAM chain weakened the planar and conjugate properties of the porphyrin molecules. Therefore, developing functionalized polymers with multiple functional properties and efficient performance is vital.

To the best of our knowledge, azobenzene, a type of conjugated molecule, has widespread application owing to its cis/trans photoisomerization under specific wavelengths of ultraviolet (UV) light (Muraoka et al., 2006), including photoelectronic techniques (Tong et al., 2006), optical data storage (Åstrand et al., 2000), and liquid crystal displays (Han and Ichimura, 2001). Several scientific studies have reported the characteristics of azobenzene–porphyrins. The results showed that the electrochemical properties of the azobenzene–porphyrins are better than simple porphyrins (Hunter and Sarson, 1996; Tse et al., 2022). Furthermore, some researchers have synthesized azobenzene-containing block copolymers with PNIPAM chains and comprehensively elucidated their thermosensitive and photosensitive properties (Irie and Kungwatchakun, 1992; Akiyama and Tamaoki, 2004). However, these multipolymers were obtained via traditional free radical copolymerization had higher polydispersity indexes (PDIs), which has bad effect on the properties of block copolymers (Ranganathan et al., 2008). Among controlled/living radical polymerization methods, ATRP has been used to synthesize some multipolymers with controllable structures and more narrow molecular weight distribution (Wang and Matyjaszewski, 1995). So, in order to achieve the polymers with low PDIs, we used ATRP to synthesize the target polymers in the present study.

First, we used zinc (II) porphyrin (ZnPor)-Br as a specific initiator to synthesize poly (6-[4-(4-methoxyphenylazo)phenoxy]hexyl methacrylate) (ZnPor-Pazo) via ATRP. Then, ZnPor-PAzo was used as a macroinitiator and polymerized with NIPAM by ATRP to obtain the photochemical and thermoresponsive diblock biomaterial (ZnPor-PAzo)–PNIPAM. By taking advantage of the excellent conjugation of polyazobenzene and thermoresponsive property of PNIPAM, we hypothesized that fluorescence and other photoactivities of the biomaterial can be improved and some new functions can be endowed. Therefore, the biomaterial will display good performance for further applications in the photochemical and biomedical fields.

## 2 Materials and methods

### 2.1 Cloud point testing

The samples were prepared as aqueous solutions (mass concentration of 2 mg/mL). These aqueous solutions were placed into the spectrophotometer cell. The samples were heated at 0.5°C/min, and the changes in the transmissivity of the aqueous solution were determined at a wavelength of 500 nm using a UV spectrophotometer.

### 2.2 Synthesis of ZnPor-PAzo via ATRP

The preparation procedures for ZnPor-OH, ZnPor-Br, and Azo are presented in the (Supplementary Schemes S1–S3) (Ringsdorf and Schmidt, 1984).

**SCHEME 1:**
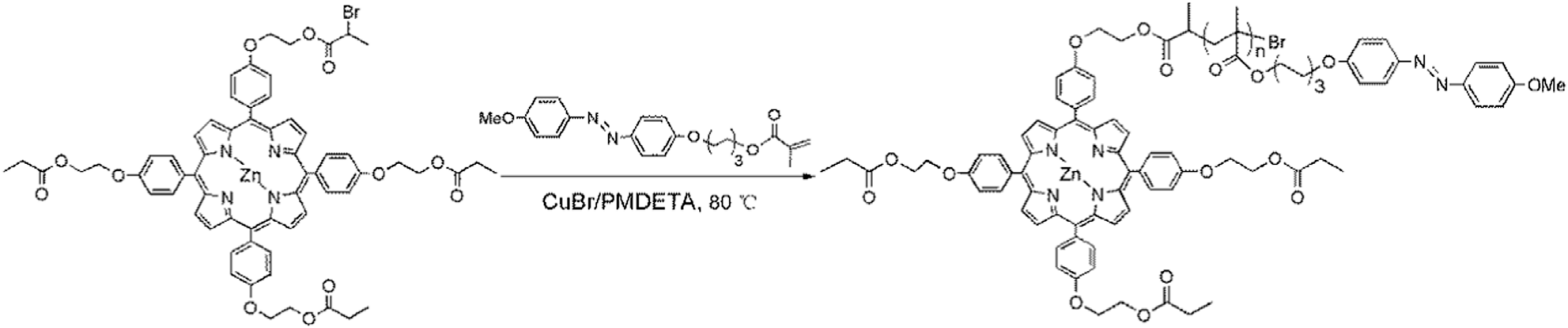
Synthesis of ZnPor-PAzo.

ZnPor-PAzo was synthesized as follows (Scheme 1). First, Azo (0.316 g, 0.8 mmol), CuBr (22 mg, 0.17 mmol), and PMDETA (40 μL, 0.17 mmol) were dissolved in anisole (2 mL) and subjected to three freeze–pump–thaw cycles under nitrogen protection. Second, ZnPor-Br (80 mg, 0.08 mmol) dissolved in anisole (2 mL) was added to the polymerization reactor to trigger the polymerization reaction. The polymerization reaction was performed for 18 h at 70°C under nitrogen protection. Finally, the copper complex from the mixed product was removed via adsorption with aluminum oxide. The obtained products were dried for 12 h to obtain clean ZnPor-PAzo. Because azobenzene contains very strong hydrophobic groups, (ZnPor-PAzo)–PNIPAMs must have relatively long hydrophilic chain segments from the PNIPAM block and a suitable length of the hydrophobic chain segment from PAzo (<10 monomer units) for good water solubility (Ravi et al., 2005). In this study, the degree of polymerization of ZnPor-PAzo was 5 (50 mg, 25% yield, *M*_n_ = 3000, PDI = 1.04).

### 2.3 Synthesis of (ZnPor-PAzo)–PNIPAM

**SCHEME 2:**
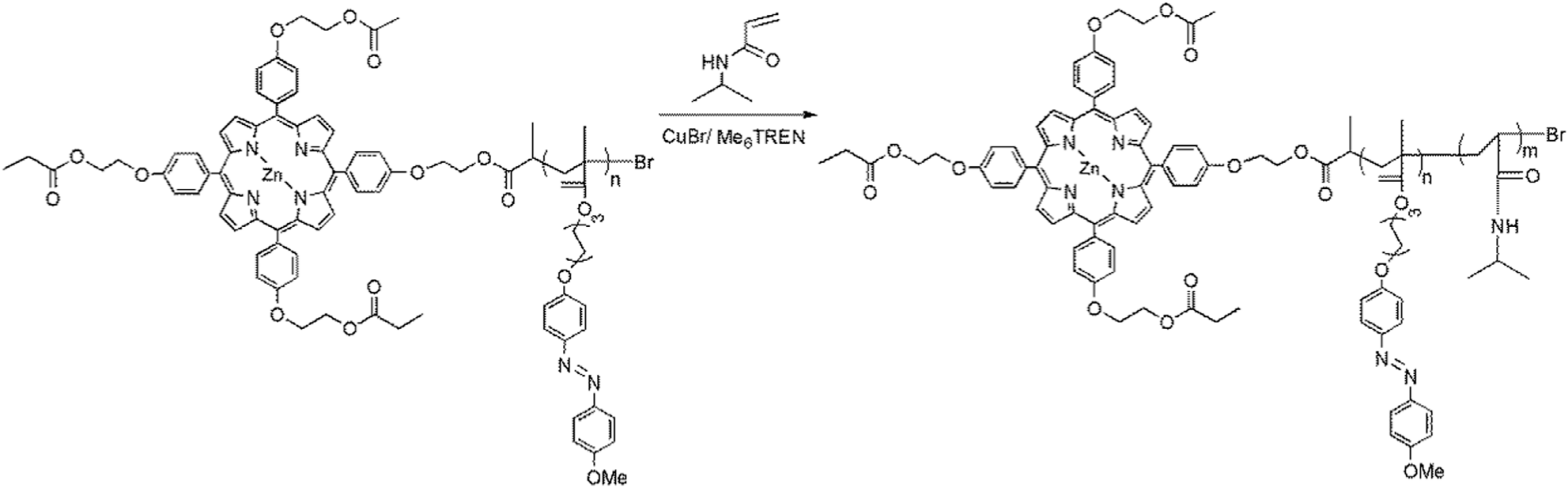
Synthesis of (ZnPor-PAzo)–PNIPAM.

(ZnPor-PAzo)–PNIPAM was synthesized as follows (Scheme 2). NIPAM (1.79 g, 0.16 mol), CuBr (16 mg, 0.08 mmol), and Me_6_TREN (20 μL, 0.08 mmol) were dissolved in a suitable mixed solvent (DMF:water = 2:1, v/v). The solution was subjected to three freeze–pump–thaw cycles under nitrogen protection. Finally, ZnPor-PAzo (*M*_n_ = 3000, 0.02 mmol, 60 mg) dissolved in DMF (0.7 mL) was added to the polymerization reactor to induce the polymerization reaction. Under nitrogen protection, the polymerization reaction was performed for 3 days at 65°C. The mixed products were purified via dialysis (MWCO, 10,000) in DMF. Finally, the dialysis products were dried for 12 h to achieve clean (ZnPor-PAzo)–PNIPAM (210 mg, 45% yield, *M*_n_ = 21,000, *M*_w_/*M*_n_ = 1.23).

### 2.4 Photocatalytic oxidative degradation test to evaluate the photocatalytic degradation of methylene blue by (ZnPor-PAzo)–PNIPAM

Hydrogen peroxide (H_2_O_2_) and (ZnPor-PAzo)–PNIPAM were added into a glass beaker containing an aqueous solution of 4 × 10^−5^ mol/L MB; this comprised the photocatalytic degradation system. The solution was stirred well in a dark environment for 20 min. Thereafter, the photocatalytic degradation system was exposed to a metal halide lamp (500 W) with an optical filter (λ > 450 nm). During the degradation process, changes in the absorbance (665 nm) were detected at regular intervals using a UV/vis spectrophotometer to determine the photocatalytic degradation of MB in real-time. The degradation rate (D) of MB was calculated using the following formula: D = (A_0_ − A)/A_0_ × 100%, where A_0_ represents the original absorption strength (665 nm) and A represents the absorption strength at a specific time during testing.

## 3 Results and discussion

### 3.1 Synthesis and characterization of (ZnPor-PAzo)–PNIPAM

(ZnPor-PAzo)–PNIPAM was prepared using two ATRP reactions (Baek et al., 2011). To obtain multipolymers, precursors with narrow molecular weight distributions must be synthesized via ATRP. In the first ATRP, ZnPor-Br was used as an initiator to polymerize ZnPor-PAzo in anisole at 80°C for 20 h. The *M*_n_ values of ZnPor-PAzo and (ZnPor-PAzo)–PNIPAM were determined using gel permeation chromatography (GPC), and the PDI was 1.04, suggesting that the sample had a narrow molecular weight distribution (Table 1). The obtained ZnPor-PAzo was then used as a macroinitiator to polymerize NIPAM to synthesize the final product [(ZnPor-PAzo)-PNIPAM]. By regulating the feeding ratio of ZnPor-PAzo to NIPAM, (ZnPor-PAzo)-PNIPAMs with different molecular weights were obtained. Table 1 presents a detailed description of the experimental conditions and physical data.

**TABLE 1:** Experimental data of the polymerization reaction and LCST of (ZnPor-PAzo)–PNIPAMs.

Samples	[M]_0_/[I]_0_ ^a^	time^d^	*M*_n(GPC)_ ^e^	*M*_n(NMR)_ ^f^	*M*_*w/*_ *M*_*n*_ ^g^	Yield (%)^h^	Azo (wt%)^i^	LCST (°C)^j^
ZnPor-(PAzo)_10_	50^b^	16	3000	3200	1.04	25	68	---
P1	200^c^	48	14,000	15,300	1.19	48	26	---
P2	400^c^	72	21,000	23,000	1.23	51	16	20
P3	600^c^	96	35,000	37,900	1.25	56	9	23
P4	800^c^	96	43,000	45,000	1.29	50	7.5	25
P5	1000^c^	96	57,000	59,700	1.32	48	3.3	28

^a^ Feed molar ratio of monomer [M]_0_ to initiator [I]_0_.

^b^ [ZnPor-Br]: [PMDETA]: [Cu(I)Br] = 1:2:2.

^c^ [ZnPor-PAzo]: [Me_6_TREN]: [Cu(I)Br] = 1:3:3.

^d^ Time of the polymerization reaction in h.

^e^ *M*_n(GPC)_ of ZnPor-PAzo, and (ZnPor-PAzo)–PNIPAMs, as determined using gel permeation chromatography (GPC).

^f^ *M*_n(NMR)_ was determined using 1H NMR, spectroscopy.

^g^ *M*_*w/*_ *M*_*n*_ of the samples prepared for GPC.

^h^ Yield of (ZnPor-PAzo)–PNIPAMs.

^i^ Proportion of ZnPor-PAzo, in (ZnPor-PAzo)–PNIPAMs.

^j^ was measured using a UV/vis spectrophotometer.

### 3.2 Fourier-transform infrared spectroscopy (FT-IR)

Supplementary Figures S7A,B illustrate the FT-IR spectra of ZnPor-PAzo and (ZnPor-PAzo)–PNIPAM, respectively. From Supplementary Figure S7A, the characteristic absorptions of PAzoMA could be clearly observed, as evidenced by the presence of a methyne stretching vibration (υ_C-H_) at 3088 cm^−1^. Furthermore, the stretching vibrations of the azo group (υ_N=N_) were observed at 1601 and 1581 cm^−1^, as demonstrated in Supplementary Figure S7A), which belong to the characteristic infrared group frequency of ZnPor-PAzo.

Supplementary Figure S7B shows FT-IR absorption spectra of PAzoMA-b-PNIPAM, there are more new characteristic absorptions than just the peaks belonging to PAzoMA. The strong absorbance at 3304 cm^−1^ was assigned to the stretching vibration (υ_N-H_) of acylamino group and the characteristic absorption peaks originated from amide group were observed at 1650 cm^−1^ (υ_C=O_) and 1550 cm^-1^ (δ_N-H_).

### 3.3 Proton nuclear magnetic resonance (^1^H NMR) studies

Supplementary Figure S8B illustrates the ^1^H NMR spectrum of ZnPor-PAzo. In contrast to the NMR spectrum of ZnPor-Br in Supplementary Figure S1A, the spectrum of ZnPor-PAzo displayed the consistent characteristic peaks belonging to ZnPor-Br at 8.77 and 8.11; the major characteristic peaks of protons belonging to PAzo were observed at 7.81, 6.91, 3.67, 1.37, and 1.30 ppm. Supplementary Figure S8A displays the ^1^H NMR spectrum of (ZnPor-PAzo)–PNIPAM. Several new proton peaks appeared at 6.31, 5.53, 4.22, 1.64, and 1.35 ppm. These characteristic signals belong to the repeat units of NIPAM. In summary, the experimental results of FT-IR and ^1^H NMR suggest the successful synthesis of end-functionalized ZnPor-PAzo and (ZnPor-PAzo)–PNIPAM via continuous ATRPs. In addition, the molecular weight of macroinitiator ZnPor-PAzo is obtained by ^1^H NMR, so the molecular weight of (ZnPor-PAzo)–PNIPAM can be acquired by calculating the integral area of peaks to methylene (f + g’) of *ZnPor-PAzo* and methyne (n) of (ZnPor-PAzo)–PNIPAM (Table 1). Furthermore, the *M*_n(GPC)_ of polymers are higher than its *M*_n(NMR)_, it is because that the molecular weights obtained from GPC using polystyrene standards are a little high.

### 3.4 GPC studies

After a series of purifications, we used the Waters e2695 GPC system (with polystyrene as the standard) to determine the *M*_n_ values of ZnPor-PAzo and (ZnPor-PAzo)–PNIPAM. Table 1 presents the test results. Supplementary Figure S9 illustrates the GPC traces. A smooth and relatively symmetric curve with only one prominent peak was observed; this indicates that ZnPor-PAzo and (ZnPor-PAzo)–PNIPAM are pure and free of any small molecule such as the initiator, monomer or other byproducts residues in the final product. Notably, the peaks of the products were relatively narrow, and the PDI (*M*_w_/*M*_n_) was 1.19–1.32, suggesting that the polymerization reactions were conducted in a controlled manner.

### 3.5 Temperature-sensitive characteristics of (ZnPor-PAzo)–PNIPAMs

**FIGURE 1:**
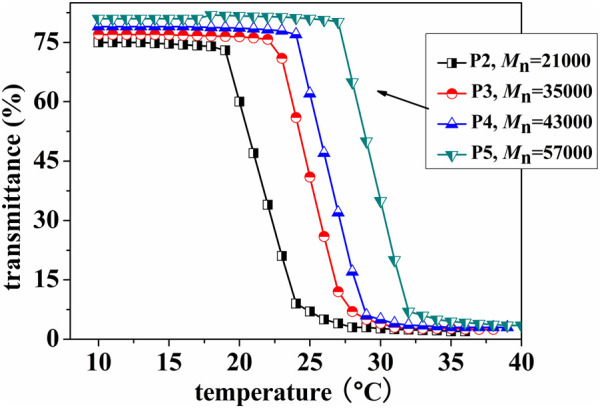
Temperature-dependent analysis of the optical transmittance of an aqueous solution of (ZnPor-PAzo)–PNIPAMs (2 mg/mL) at a wavelength of 500 nm.

To investigate the temperature-sensitive characteristics of (ZnPor-PAzo)–PNIPAMs, the lower critical solution temperature (LCST) was determined based on the optical transmissivity of (ZnPor-PAzo)–PNIPAM using the UVmini-1240 spectrophotometer (Shimadzu). Figure 1 shows the temperature-dependent analysis of the optical transmissivity of an aqueous solution of (ZnPor-PAzo)–PNIPAM at a wavelength of 500 nm. In the first stage, there was no marked variation in optical transmissivity with increasing temperature. This experimental phenomenon indicates that the temperature at this stage is below the LCST and that the hydrogen bond between the water molecule and amino group plays a prominent role; therefore, they are highly soluble in water. However, as the temperature increased, the powerful associative interaction of hydrophilic isopropyl inhibited the formation of intermolecular hydrogen bonds; as a result, the powerful repulsion arising from the hydrophobic groups led to the formation of large aggregates; the solution gradually became turbid when the temperature exceeds the LCST. In this experiment, the temperature at which the transmittance of (ZnPor-PAzo)–PNIPAM decreased by >10% was defined as the LCST at 500 nm. The results are listed in Table 1. As predicted, the results of the LCST experiment agreed with the finding that hydrophobic groups decrease the LCST of PNIPAM. The LCST of (ZnPor-PAzo)–PNIPAMs were lower than that of the homopolyme NIPAM, and the (ZnPor-PAzo)–PNIPAMs with the*M*_n(NMR)_ of 15,300 cannot show the thermo-responsive property owing to the critical effect of the hydrophobic grouping ZnPor-PAzo (Xia et al., 2006). Furthermore, the LCST of (ZnPor-PAzo)–PNIPAMs gradually increased with an increase in *M*_n_. The primary reason for this is that the effect of the hydrophilic groups on the properties of (ZnPor-PAzo)–PNIPAMs correspondingly increased.”

### 3.6 Photoresponsive property of (ZnPor-PAzo)–PNIPAMs

**FIGURE 2:**
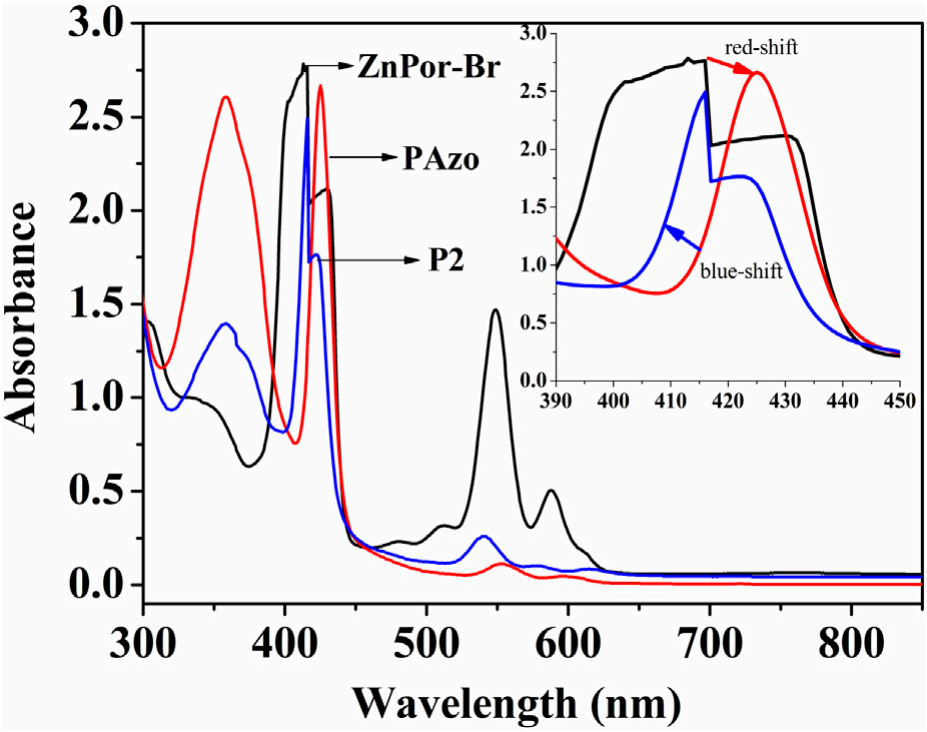
Ultraviolet/visible spectra of ZnPor-Br, ZnPor-Pazo, and (ZnPor-PAzo)–PNIPAMs (*M*_n_ = 21,000) in CH_2_Cl_2_ (0.4 mmol/L).

Figure 2 intuitively displays the UV/vis spectra of ZnPor-Br, PAzoMA, and (ZnPor-PAzo)-PNIPAMs in the form of a superposition. The B- and Q-bands of ZnPor-Br were located at 415 and 545–590 nm, respectively; this finding is consistent with that of similar metalloporphyrin derivatives (Papkovsky and O’riordan, 2005). Meanwhile, the UV/vis absorption spectrum of ZnPor-PAzo revealed the characteristic band of azobenzene at approximately 360 nm and the B and Q-bands at 424 and 545–590 nm, receptively. Compared with ZnPor-Br, the B- and Q-bands of ZnPor-PAzo exhibited a bathochromic shift. This is because the organic conjugated groups (azobenzene) replace the electron-withdrawing groups (-Br), thereby decreasing the lowest empty orbital and increasing the highest orbital energy levels simultaneously; therefore the B- and Q-bands exhibit a redshift phenomenon. Moreover, we observed another interesting phenomenon: compared with ZnPor-PAzo the B- and Q-bands of (ZnPor-PAzo)–PNIPAMs exhibited a blue shift, which was caused by the severe steric hindrance effect of the polymer segments.

### 3.7 Cis/trans isomerization of (ZnPor-PAzo)–PNIPAMs under UV/vis light irradiation

**FIGURE 3:**
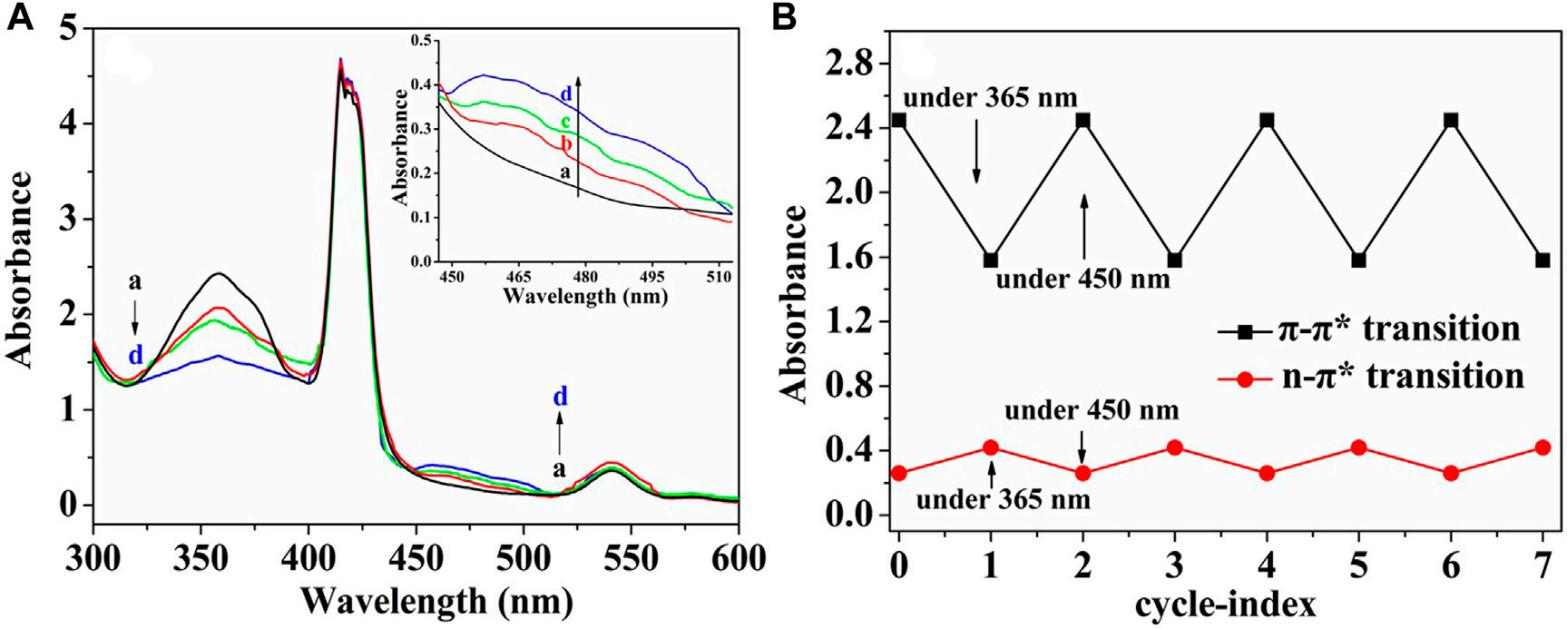
**(A)** Spectral changes in the aqueous solution of (ZnPor-PAzo)–PNIPAM (*M*_n_ = 21,000) under ultraviolet UV light (365 nm, 8 W). Exposure time: (a) 0 min, (b) 20 min, (c) 50 min, and (d) 90 min. **(B)** Repetitive cycle experiment of the cis/trans isomerization of (ZnPor-PAzo)–PNIPAM (*M*_n_ = 21,000). The biomaterial was UV irradiated for 90 min and exposed to visible light (450 nm) for 60 min for one operating cycle.

The cis/trans isomerization of (ZnPor-PAzo)–PNIPAM was determined in aqueous solutions after UV exposure (365 nm, 8 W). Figure 3 illustrates the changes in the UV/vis absorption spectra at different UV illumination times. (ZnPor-PAzo)–PNIPAM exhibited two absorption bands at approximately 360 and 470 nm; these bands belonged to the π-π* and n–π* transition bands of trans- and cis-azobenzene, respectively. With an increase in UV irradiation time, the intensity of the π-π* transition band at approximately 360 nm gradually became weak; on the other hand, the n–π* transition band at approximately 470 nm was gradually enhanced. The photostationary state was achieved at 365 nm after irradiation for 90 min. When the (ZnPor-PAzo)–PNIPAM was exposed to visible light (450 nm) or dark conditions for approximately 60 min, the trans-azobenzene content of (ZnPor-PAzo)–PNIPAM was completely recovered. Furthermore, a repetitive cycle experiment of the cis/trans isomerization of (ZnPor-PAzo)–PNIPAM was performed; the results are illustrated in Figure 3A. After irradiation for 90 min at 365 nm, the intensity of the n–π* transition band at approximately 470 nm was the highest and that of the π-π* transition band at approximately 360 nm was the lowest. In contrast, after light irradiation for 60 min at 450 nm, the intensity of the n–π* transition band was the lowest, whereas that of the π-π* transition band was the highest. These steps were repeated seven times. The cis/trans isomerization was stable and did not delay the reaction time. Taken together, the above experimental results suggest that (ZnPor-PAzo)–PNIPAM not only undergoes cis/trans isomerization but also exhibits favorable repeatability, which is vital for practical applications.

### 3.8 Fluorescence spectrum results

**FIGURE 4:**
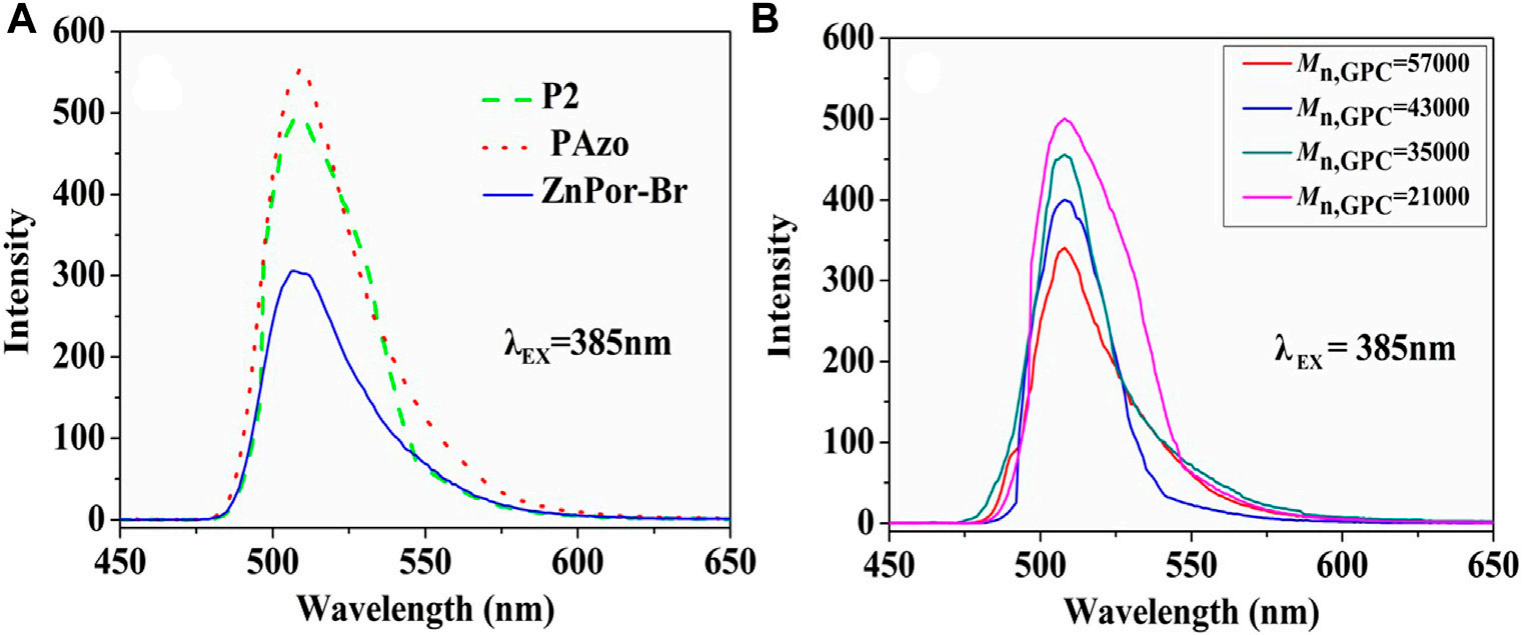
**(A)** Fluorescence spectra of ZnPor-Br, ZnPor-PAzo, and P2 [(ZnPor-PAzo)–PNIPAM, *M*_n_ = 21,000) at a concentration of 2 × 10^−5^ mol/L in DMF. Analysis was performed using the Shimadzu RF-5301PC fluorescence spectrophotometer **(B)** Changes in the fluorescence intensity of (ZnPor-PAzo)–PNIPAMs with different *M*_n_.

Several related experimental studies have reported that porphyrins and their derivatives can be used as fluorescent dyes (Fang et al., 2008; Tao et al., 2011). Therefore, we discussed and compared the fluorescence properties of ZnPor-Br, ZnPor-PAzo, and (ZnPor-PAzo)–PNIPAM. Figure 4A illustrates the fluorescence spectra of the samples. The emission maximum was observed at approximately 507 nm at an excitation wavelength of 385 nm. However, the fluorescence intensities of ZnPor-Br, ZnPor-PAzo, and (ZnPor-PAzo)–PNIPAM were significantly different. The fluorescence intensity of ZnPor-PAzo was much stronger than that of ZnPor-Br. This may be because of the following two points. First, ZnPor-PAzo has a noticeable absorption peak between 335 and 390 nm, which has some degree of overlap with the PLE of ZnPor-PAzo (385 nm), and ZnPor-PAzo achieves more energy because of energy transmission. Second, the addition of azobenzene may considerably enhance the π-electronic conjugation. In contrast, the fluorescence intensity of (ZnPor-PAzo)–PNIPAM was weaker than that of ZnPor-PAzo to some extent. This could be because the π-π conjugation was weakened by the severe steric hindrance effect of the polymer segments. In addition, we studied the effect of Mn on the fluorescence intensity of (ZnPor-PAzo)–PNIPAM at an excitation wavelength of 385 nm. As shown in Figure 4B, the fluorescence intensity of (ZnPor-PAzo)–PNIPAM slowly decayed with an increase in Mn, without significant fluorescence quenching after polymerization. (Qiu et al., 2014).”

### 3.9 Photocatalytic performance of (ZnPor-PAzo)–PNIPAM on MB

**FIGURE 5:**
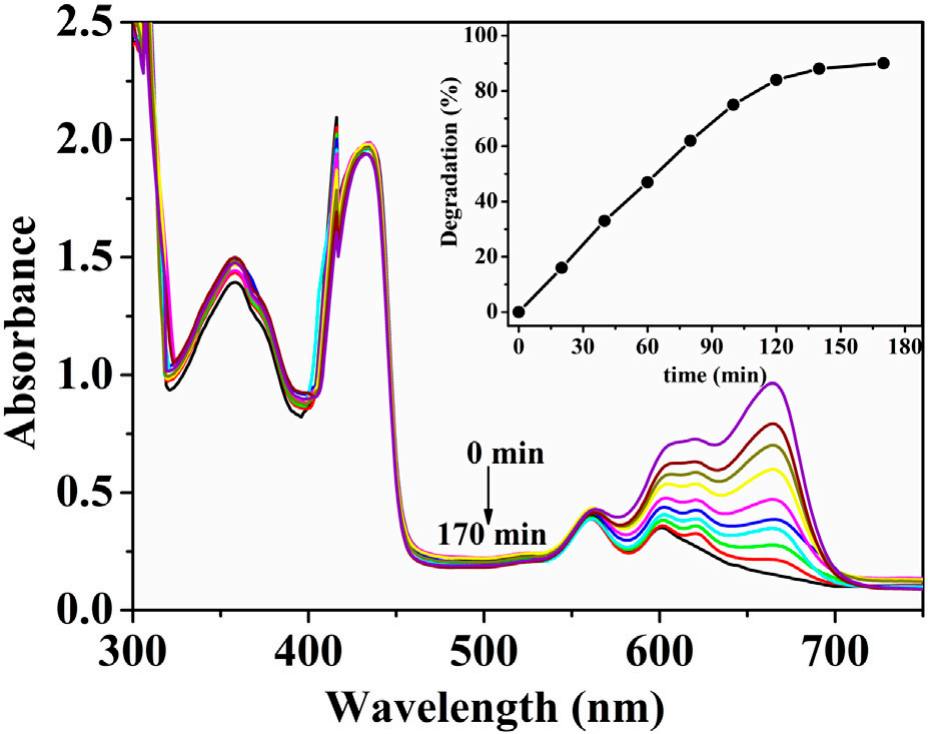
Changes in the ultraviolet (UV)/vis spectral absorbance of an aqueous solution of 4 × 10^−5^ mol/L methylene blue [MB] at 665 nm at regular time intervals at 19.5°C. [(ZnPor-PAzo)–PNIPAM (*M*_n_ = 21,000)] = 2 × 10^−5^ mol/L, [H_2_O_2_] = 40 mL/L, pH = 2, and λ > 450 nm.

In this study, the photocatalytic activity of (ZnPor-PAzo)–PNIPAM was investigated by performing photocatalytic experiments in which MB was exposed to visible light. After a series of photocatalytic experiments, we verified that the experimental conditions and factors influencing the photocatalytic activity of (ZnPor-PAzo)–PNIPAM were consistent with those reported in our previous study (Faustova et al., 2020). The experimental results are shown in Figure 5; the characteristic peak of MB at 665 nm rapidly decreased with increasing reaction time and nearly disappeared after 170 min. In other words, MB concentration gradually decreased as the earlier catalytic degradation reaction progressed; the degradation rate reached approximately 90%. The above experimental results suggest that (ZnPor-PAzo)–PNIPAM exerts an excited photocatalytic degradation effect on MB under visible light irradiation. Furthermore, the peaks of (ZnPor-PAzo)–PNIPAM at 360 and 420 nm did not change as degradation time increased; this suggests that (ZnPor-PAzo)–PNIPAM maintains structural stability during catalytic degradation.

**FIGURE 6:**
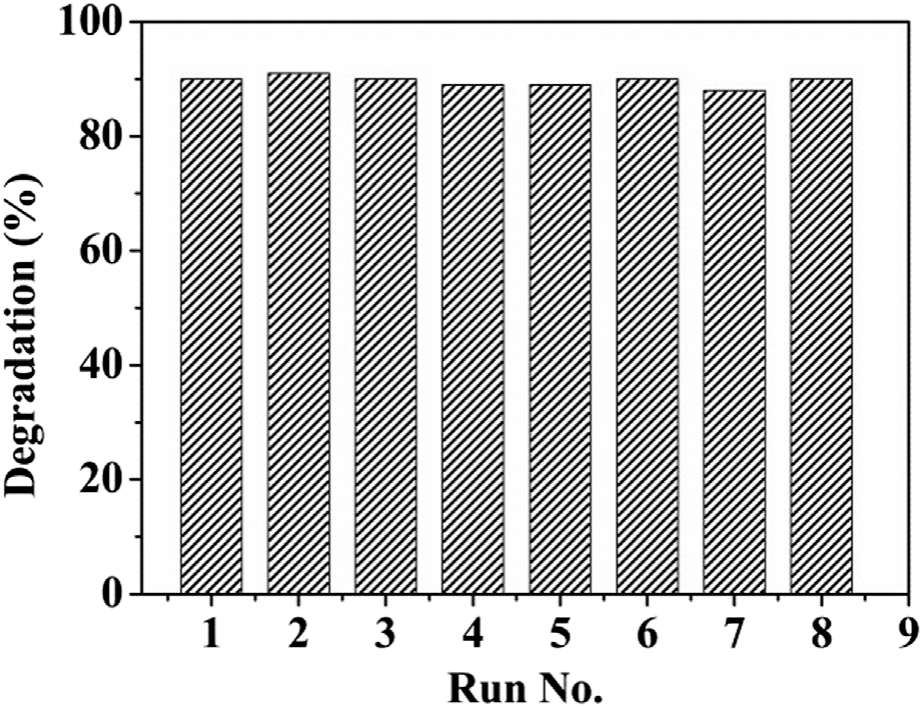
Recycling and reusing experiments of (ZnPor-PAzo)–PNIPAM for the photocatalytic oxidative degradation of methylene blue (MB). [MB] = 4 × 10^−5^ mol/L, [(ZnPor-PAzo)–PNIPAM (*M*_n_ = 21,000)] = 2 × 10^−5^ mol/L, [H_2_O_2_] = 40 mL/L, and pH = 2.

Finally, the reusability and stability of (ZnPor-PAzo)–PNIPAM were tested. After the catalytic degradation of MB with the fresh (ZnPor-PAzo)–PNIPAM catalyst, the used (ZnPor-PAzo)–PNIPAM can be recycled via a simple and easy filtration process owing to its thermoresponsive properties. First, the temperature was increased above the LCST; (ZnPor-PAzo)–PNIPAM agglomerated to form solid particles in water. The precipitated solid particles were then filtered and washed five times with deionized water. Finally, the solid particles were dried at 60°C in vacuum for 10 h. Recycled (ZnPor-PAzo)–PNIPAM was added again to the newly prepared MB solution, and a second catalytic degradation and recovery experiment was performed under invariable experimental conditions. Recycling tests were performed eight times. The results of the tests are displayed in Figure 6. Notably, the degradation rate of MB basically remained at approximately 90%. The abovementioned experimental results suggest that (ZnPor-PAzo)–PNIPAM exhibits excellent photocatalytic degradation ability and can be easily recycled without secondary pollution.

## 4 Conclusion

By the ATRP reactions, we have successfully fabricated a series of efficient and green photocatalytic degradation agents [(ZnPor-PAzo)–PNIPAM] for MB under visible light. In addition, (ZnPor-PAzo)–PNIPAM can be recycled several times to maintain its photocatalytic degradation characteristics. The thermoresponsive diblock copolymers (ZnPor-PAzo)–PNIPAM not only exhibited representative cis/trans isomerism when exposed to sufficient UV light but also had significantly enhanced fluorescence intensity in DMF. Therefore, these (ZnPor-PAzo)-PNIPAMs which have good temperature-sensitive water solubility and fluorescence characteristic expected to have potential application value in the field of biomolecular recognition.

## Data Availability

The original contributions presented in the study are included in the article/Supplementary Material, further inquiries can be directed to the corresponding author.
