# Supplementary material for: Synthesis of a novel photochemical and thermoresponsive diblock biomaterial with end-functionalized zinc porphyrin
Source: Front Bioeng Biotechnol. 2023 Dec 1;11:1268458. doi: 10.3389/fbioe.2023.1268458 (PMC10722221; doi:10.3389/fbioe.2023.1268458)
Supplement: Supplementary file 1 [file Presentation1.pdf]

1. Synthesis of p-hydroxyethoxybenzaldehyde

2.3 g (0.1 mol) Sodium Metal was cut into small pieces then added into 90 mL ethanol quickly at reflux temperature. Under the protection of nitrogen, 12.2 g (0.1 mol) p-hydroxybenzaldehyde was added into alcohol sodium solution with stirring 15 min. 7.5 mL 2-bromoethyl alcohol was slowly added into reaction vessel while stirring. The mixture was reacted for 5-6 h at reflux temperature, then evaporated to obtain pale yellow oily matter. The obtained mixture was purified by column chromatography (SiO_2, petroleum ether/ ethyl acetate 3:1 v/v) to obtain a purple p-hydroxyethoxybenzaldehyde(**Scheme S1**).

FT-IR: 3400 cm^{-1}(ν_{-OH}), 2850～2925 cm^{-1}($\nu_{-CH2,\ -CH3}$)[**Figure S1(a)**]. ^{1}H NMR:9.86 (S, 1H), 7.83 (d, 2H), 7.00(d, 2H), 4.17(t, 2H,), 4.00(t, 2H), 2.87(d, 1H) [**Figure S2(A)**]. (^{1}H NMR spectra were carried out on a Bruker Avance 400MHz spectrometer at ambient temperature, using $CDCl_3$ or DMSO-d_6 as solvent, TMS for ^{1}H calibration. FT-IR spectra were recorded on a FT-IR 8400S (Shimadzu) spectrometer. The spectra were collected at 40 scans with a resolution of 4 cm^{-1})

2. Synthesis of 5,10,15,20-tetra(p-hydroxyethylphenyl) porphyrin tripropionate (Por-OH)

120 mL (0.1 mol) propionic acid was added into reaction vessel, heated to return reflux temperature. 12 g (0.072 mol) p-hydroxyethoxybenzaldehyde was added into propionic acid with stirring, 5.6 mL (0.072 mol) new steamed pyrrole was added into reaction vessel dropwise while stirring. The mixture was reacted for 50 min at reflux temperature, then added 140 mL methanol standing crystallization in refrigerator. The obtained crystal was purified by column chromatography (SiO_2, dichloromethane/methanol 50:1 v/v) to obtain purple Por-OH (**Scheme S1**).

FT-IR: 3430 cm^{-1}(ν_{-OH}), 2850 ～ 2960 cm^{-1}($\nu_{-CH2,\ -CH3}$), 3430 cm^{-1}(ν_{-OH}), 1750 cm^{-1}($\nu_{C=O}$), 729 cm^{-1}, 810 cm^{-1}, 990 cm^{-1} are the skeletal vibration of pyrrole, 3317 cm^{-1}(ν_{N-H})、969 cm^{-1}(δ_{N-H}) [**Figure S1(b)**]. ^{1}H NMR: 8.86(s, 8H)，8.11(d, 8H), 7.27(d, 8H)，4.61(t, 6H)，4.44(m, 6H)，4.35(t, 2H), 4.14(t, 6H), 2.50(q, 6H), 2.28(s, 1H), 1.27(t, 9H)，-2.75(s, 2H) [**Figure S2(B)**]. Anal. Calcd for $C_{62}H_{59}N_4O_{11}$: C, 70.61; H,

5.49; N, 5.54. Found: C, 71.61; H, 5.71; N, 5.48.(Element analysis was obtained on a Carlo Erba-MOD1106 instrument.)

3. Synthesis of 5, 10, 15, 20-tetra(p-hydroxyethylphenyl) zincporphyrin tripropionate (ZnPor-OH)

100 mg (0.15mol) 5, 10, 15, 20-tetra(p-hydroxyethylphenyl) porphyrin tripropionate was added into DMF, heated to return reflux temperature. 80 mg (0. 36mol) zinc acetate with stirring at reflux temperature reacted for 2 h. subsequently, the mixed solution was evaporated most DMF under vacuum, then added into 150 mL deionized water, the zinzolin solid precipitated, filtered and dried at 65°C under vacuum (**Scheme S1**).

FT-IR: 3430 cm^{-1}(ν_{-OH}), 1750 cm^{-1}($\nu_{C=O}$), 2850～2925 cm^{-1}($\nu_{-CH_2, -CH_3}$)，729 cm^{-1}, 810 cm^{-1}, 960 cm^{-1}, 990 cm^{-1} are the skeletal vibration of pyrrole, 1000 cm^{-1}($\delta_{Zn\text{-}N}$) [**Figure S1(c)**]. ^{1}H NMR: 8.86 (s, 8H)，8.11 (d, 8H), 7.27 (d, 8H)，4.61 (t, 6H)，4.44 (m,6H)， 4.35 (t, 2H), 4.14 (t, 6H), 2.50 (q, 6H)， 2.28 (s, 1H), 1.27 (t, 9H)[**Figure S2(C)**]. Anal. Calcd for $C_{62}H_{59}N_4O_{11}Zn$: C, 67.61; H, 5.40; N, 5.09. Found: C, 67.93; H, 5.37; N, 4.59.

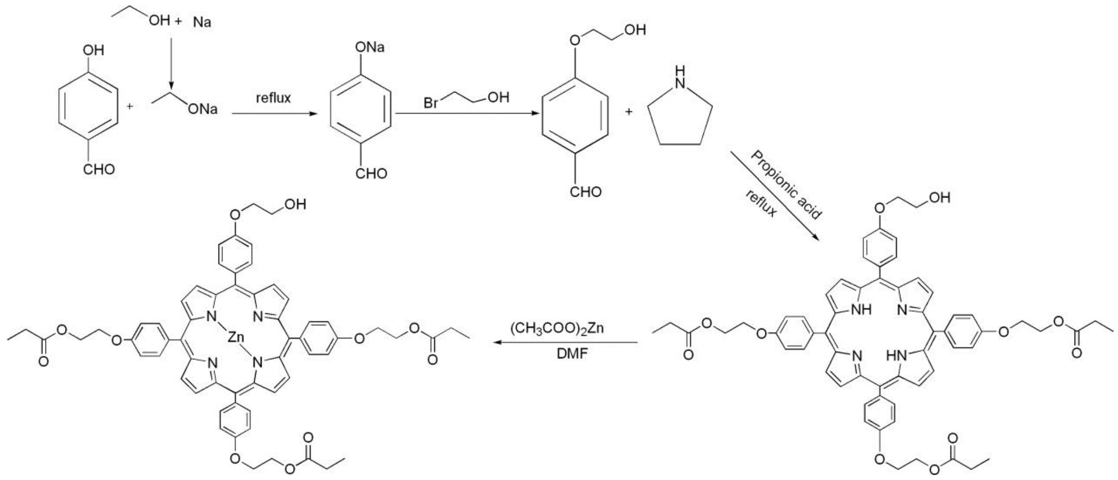

Scheme S1 Synthetic route of 5, 10, 15, 20-tetra(p-hydroxyethylphenyl) zincporphyrin tripropionate.

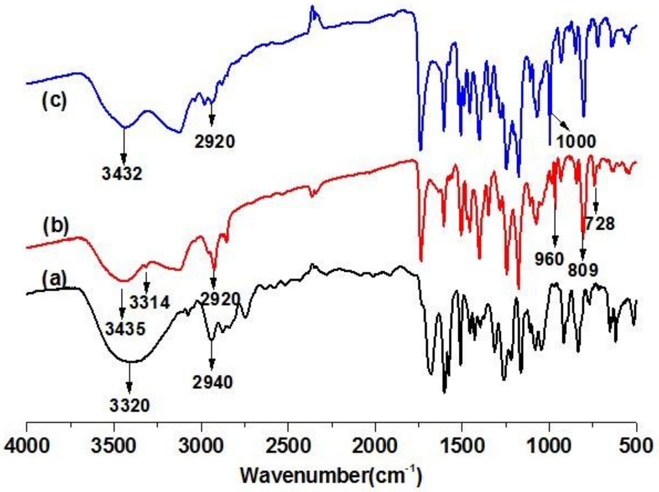

Figure S1. FT-IR spectra of (a) p-hydroxyethoxybenzaldehyde, (b) Por-OH, (c) ZnPor-OH

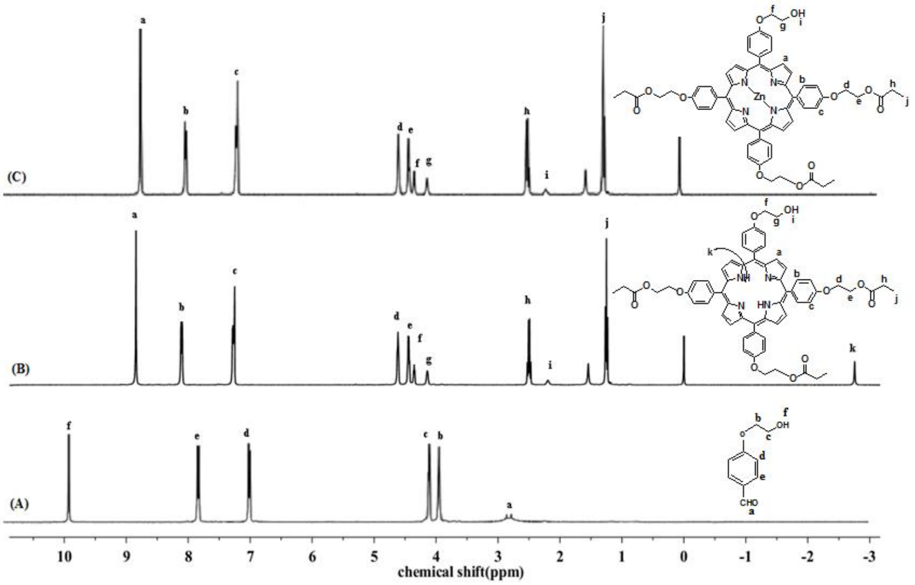

Figure S2. ^{1}H NMR spectra of (A) p-hydroxyethoxybenzaldehyde, (B) Por-OH, (C) ZnPor-OH

4. Synthesis of 5, 10, 15, 20-tetra(p-bromopropanoyloxyethylphenyl) zincporphyrin tripropionate(ZnPor-Br)

5, 10, 15, 20-tetra(p-hydroxyethylphenyl) zincporphyrin tripropionate (100 mg, 0.1mmol) was added into dichloromethane cooled to 0 °C in an ice bath, and then α-bromopropionyl bromide (0.25 mL, 0.2 mmol) and triethylamine(0.28 mL, 0.2 mmol) was added dropwise. After being stirred for 1 h at 0 °C, the solution was stirred further for 20 h at room temperature. Subsequently, the mixed solution was purified by extraction-separation. The organic layer was dried over Na_2SO_4, filtered,

and then evaporated to obtain zinzolin solid (**Scheme S2**).

FT-IR: 2850～2925 cm^{-1}($\nu_{-CH_2, -CH_3}$), 1730 cm^{-1}($\nu_{C=O}$), 729 cm^{-1}, 810 cm^{-1}, 960 cm^{-1}, 990 cm^{-1} are the skeletal vibration of pyrrole, 1000 cm^{-1}(δ_{Zn-N}) [**Figure S3**]. ^{1}H NMR: 8.85 (s, 8H), 8.13 (d, 8H), 7.30 (d, 8H), 4.73 (s, 2H), 4.63 (s, 6H), 4.58 (d, 1H), 4.53 (d, 6H), 4.49 (d, 6H),2.53~2.48 (q, 6H), 1.83 (d, 3H) ,1.24 (t, 9H) [**Figure S4.**]. Anal. Calcd for $C_{64}H_{59}BrN_4O_{12}Zn$: C, 62.83; H, 4.77; N, 4.40. Found: C, 62.92; H, 4.87; N, 4.58.

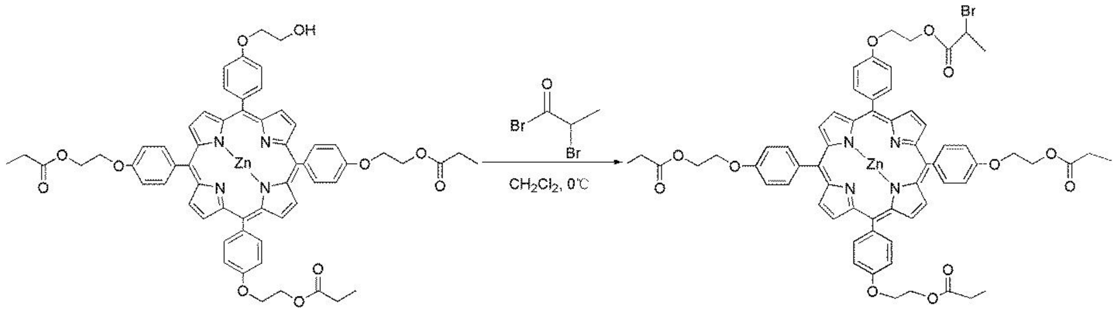

Scheme S2. Synthetic route of ZnPor-Br.

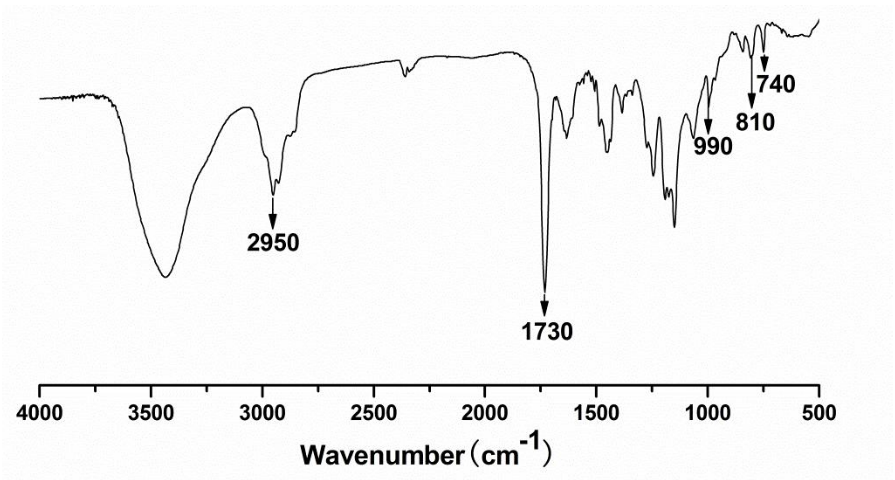

Figure S3. FT-IR spectra of ZnPor-Br.

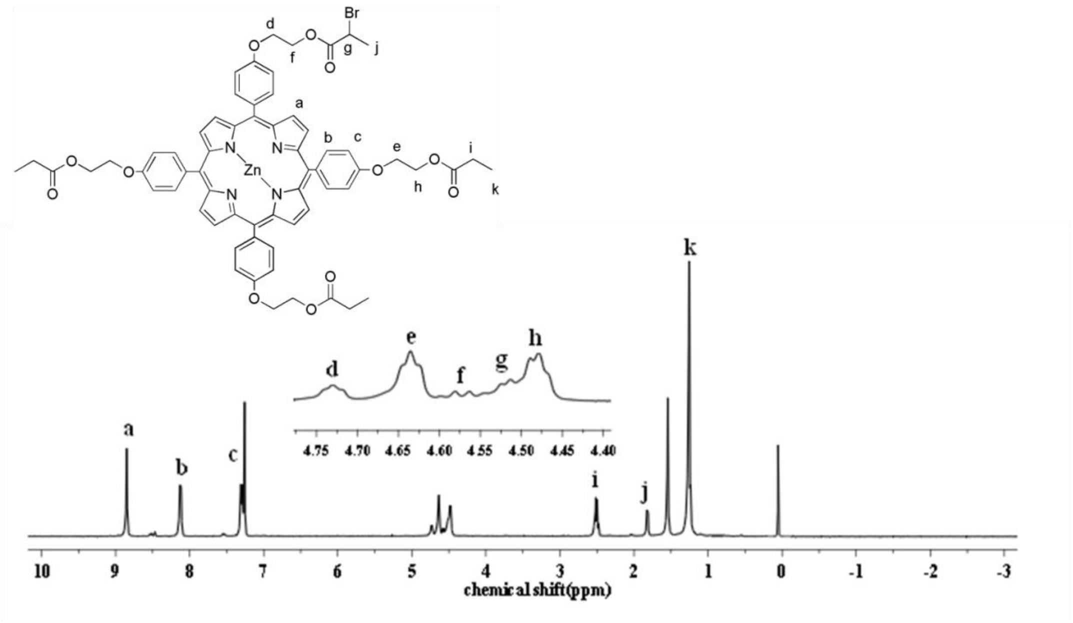

Figure S4. ^{1}H NMR spectra of ZnPor-Br.

5. Synthesis of 6-[4-(4-methoxyphenylazo)phenoxy]hexylmethacrylate (AzoMA)

The monomer, 6-[4-(4-methoxyphenylazo)phenoxy]hexyl methacrylate(AzoMA), was synthesized following a previous method (***Scheme S3***). FT-IR: 2850～2950 cm^{-1}($\nu_{-CH_2,\ -CH_3}$), 1715 cm^{-1}($\nu_{C=O}$), 1650 cm^{-1}($\nu_{C=C}$), 1600, 1585 cm^{-1} ($\nu_{N=N}$), 725 cm^{-1}(δ_{-CH_2-}) [**Figure S5.**]. ^{1}H NMR($CDCl_3$, ppm): 7.87 (m, 4H), 6.99 (m,4H), 6.10 (s, 1H), 5.55 (s, 1H), 4.17 (t, 2H), 4.04 (t, 2H), 3.88 (s,3H), 1.95 (s, 3H), 1.87–1.80 (m, 2H), 1.77-1.70(m, 2H), 1.58-1.45(m, 4H) [**Figure S6.**].

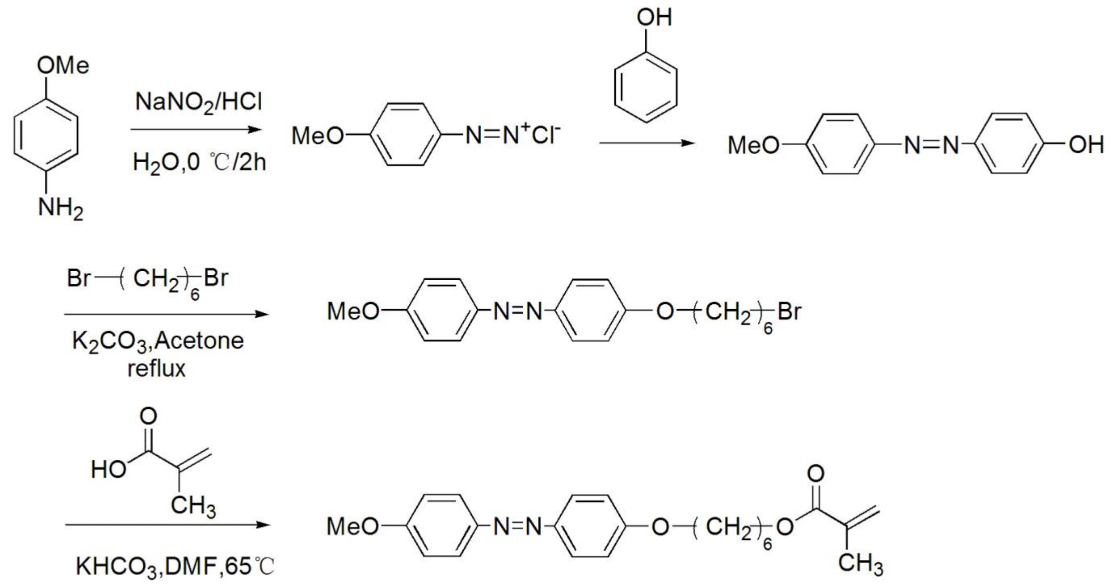

Scheme S3. Synthetic route of the monomer AzoMA.

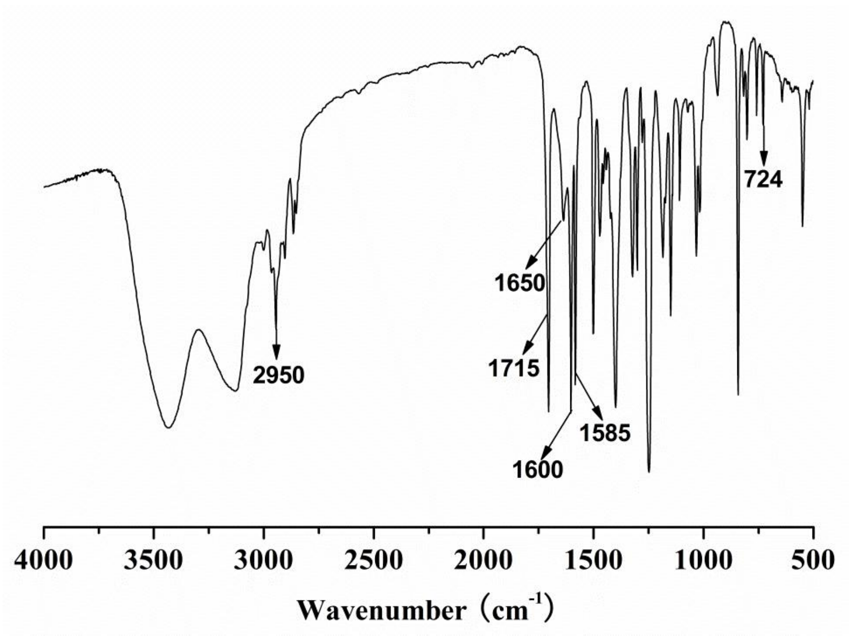

Figure S5. FT-IR spectra of AzoMA.

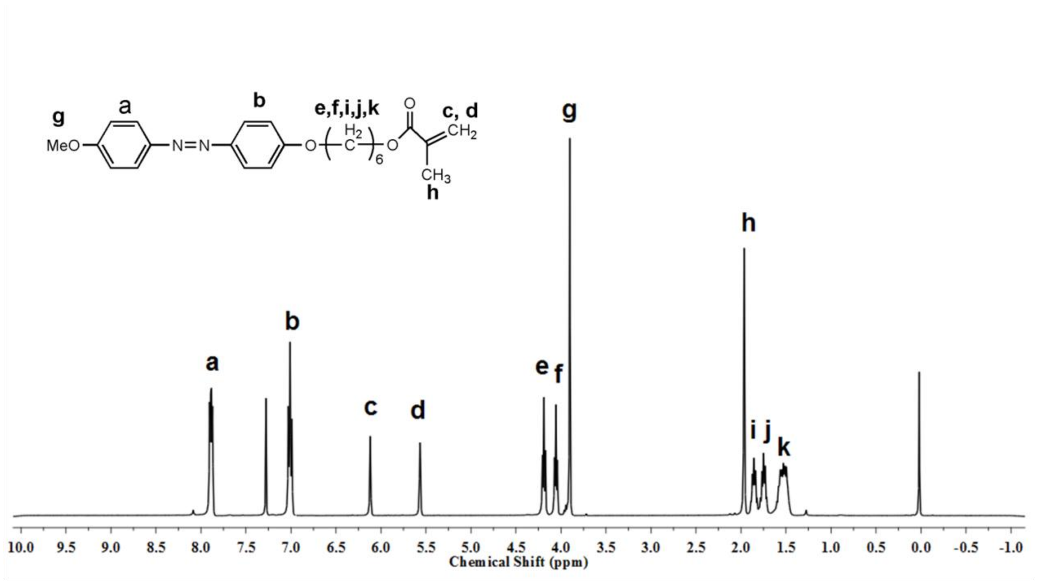

Figure S6. ^{1}H NMR spectra of AzoMA.

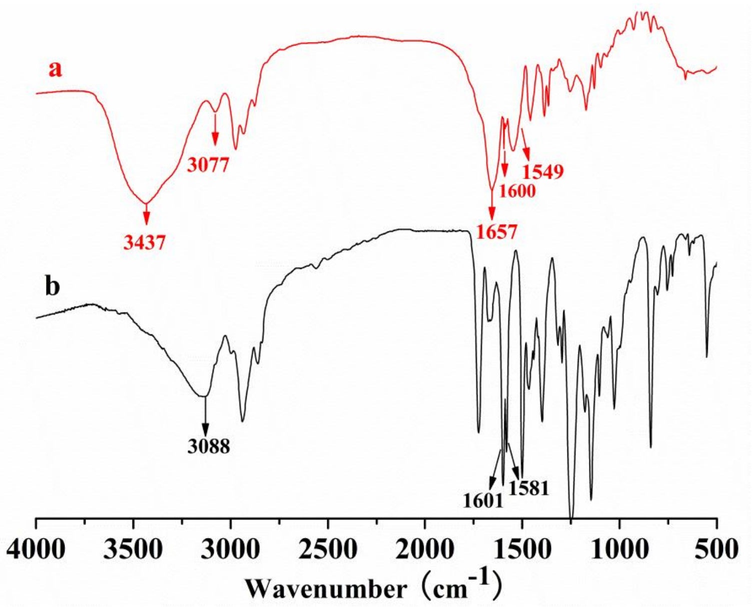

Figure S7. FT-IR spectra of (ZnPor-PAzo)-PNIPAM (a) and ZnPor-PAzo (b)

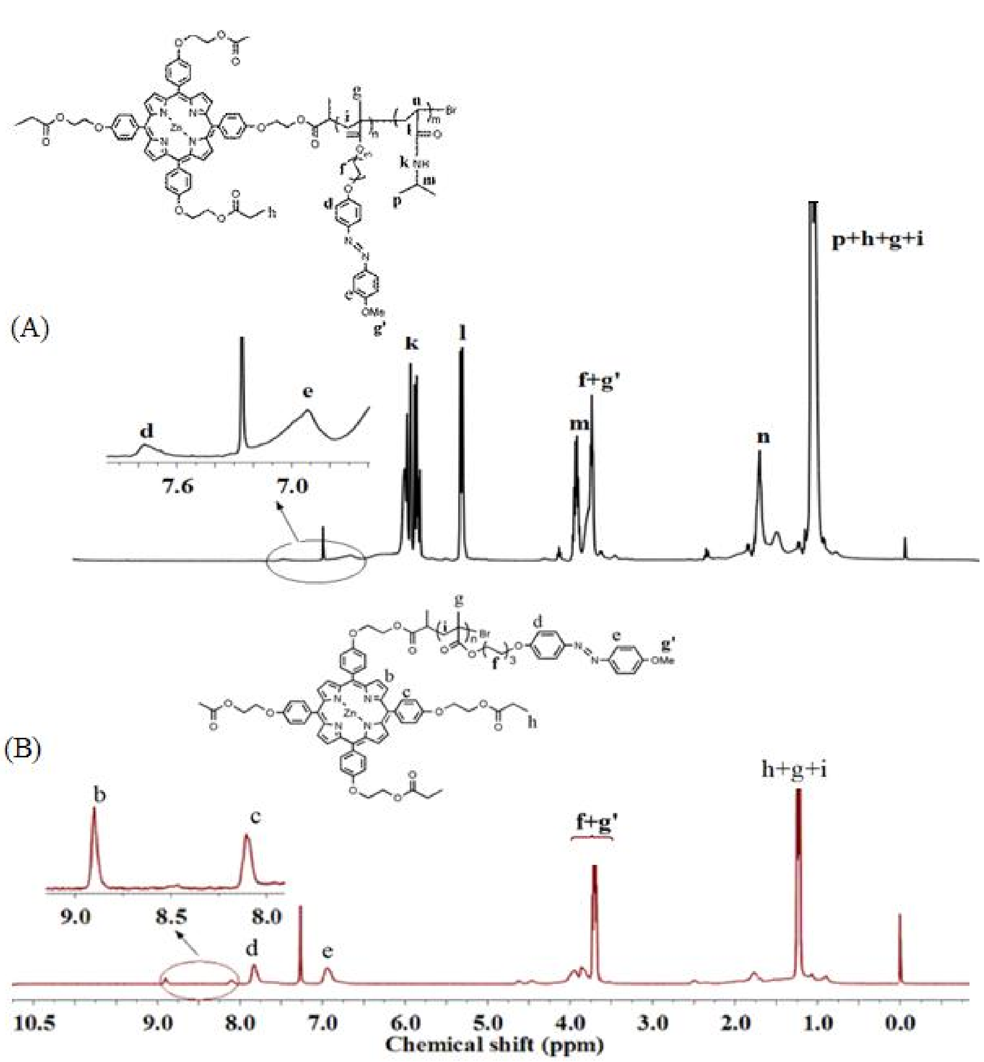

Figure S8. ^{1}H NMR spectra of (ZnPor-PAzo)-PNIPAM (A) and ZnPor-PAzo (B)

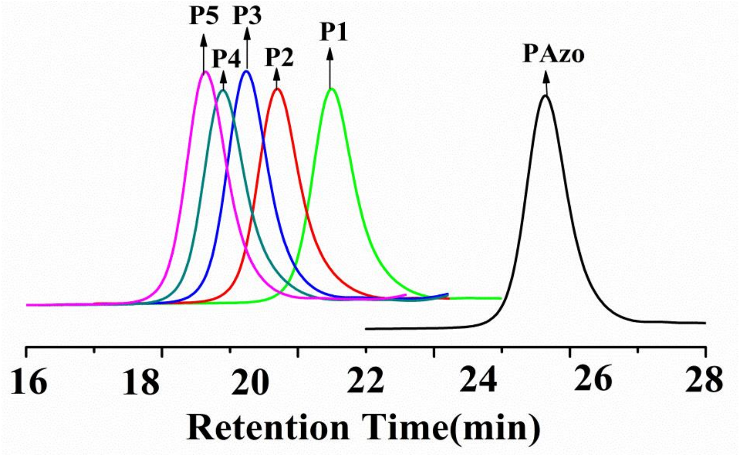

Figure S9. Gel permeation chromatography (GPC) traces of ZnPor-PAzo and (ZnPor-PAzo)–PNIPAMs.
